# Primary Bronchial Hybrid Low‐Grade Fibromyxoid Sarcoma/Sclerosing Epithelioid Fibrosarcoma With *EWSR1::CREB3L1* Fusion: Diagnostic Insights From a Challenging Case in the Respiratory Tract

**DOI:** 10.1111/pin.70185

**Published:** 2026-09-25

**Authors:** Hiroko Ikeda, Seiichi Kakegawa, Daisuke Saito, Keita Kinoshita, Ayumi Ito, Keishi Mizuguchi, Seiji Yano, Tomohiro Kobayashi, Satoshi Kobayashi, Akihiro Nishiyama, Shinji Takeuchi, Isao Matsumoto

**Affiliations:** ^1^ Department of Diagnostic Pathology Kanazawa University Hospital Kanazawa Japan; ^2^ Department of Thoracic Surgery Kanazawa University Kanazawa Japan; ^3^ Department of Respiratory Medicine Kanazawa University Kanazawa Japan; ^4^ Department of Radiology Kanazawa University Graduate School of Medicine Kanazawa Japan; ^5^ Department of Medical Oncology Kanazawa University Hospital Kanazawa Japan

**Keywords:** Bronchial sarcoma, *EWSR1::CREB3L1* fusion, hybrid LGFMS/SEF, low‐grade fibromyxoid sarcoma, MUC4, respiratory tract, sclerosing epithelioid fibrosarcoma

## Abstract

Hybrid low‐grade fibromyxoid sarcoma/sclerosing epithelioid fibrosarcoma (LGFMS/SEF) is an ultra‐rare fibroblastic sarcoma that typically arises in the soft tissues. Primary involvement of the respiratory tract is extremely uncommon, and, to our knowledge, no cases arising in the bronchial wall have previously been reported. We describe a hybrid LGFMS/SEF arising in the right bronchus of a Japanese woman in her 40 s. The 2‐cm tumor was incidentally detected, and imaging studies demonstrated no other lesions. Histologically, the tumor consisted predominantly of bland spindle cells within fibromyxoid stroma with collagen rosettes, admixed with epithelioid fibroblasts arranged in cords. Immunohistochemically, the tumor showed diffuse positivity for MUC4. Comprehensive genomic profiling performed for diagnostic purposes identified an *EWSR1::CREB3L1* fusion gene, supporting the diagnosis of hybrid LGFMS/SEF. Retrospective review of the transbronchial biopsy specimen revealed subtle spindle cells showing MUC4 expression by immunohistochemistry and *EWSR1* rearrangement by fluorescence in situ hybridization, which had initially been interpreted as inflammatory change. This case highlights important diagnostic pitfalls of hybrid LGFMS/SEF and provides the clinicopathological insights into *EWSR1::CREB3L1*‐associated fibroblastic sarcoma arising in the bronchial wall.

## Graphical abstract

We report a primary bronchial hybrid low‐grade fibromyxoid sarcoma/sclerosing epithelioid fibrosarcoma (LGFMS/SEF) characterized by histological, immunohistochemical, and molecular findings, including detection of an *EWSR1::CREB3L1* fusion by comprehensive genomic profiling. We highlight important diagnostic pitfalls and discuss the clinicopathological and molecular features of this rare fibroblastic sarcoma of the respiratory tract.

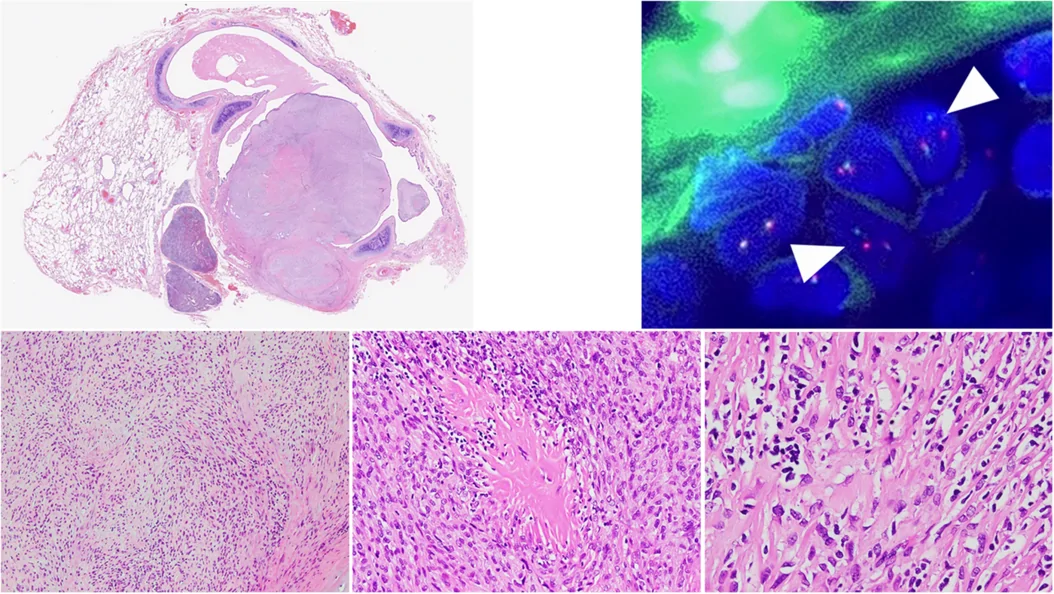

**Abbreviations**
CT: Computed tomography
FDG‐PET: Fluorodeoxyglucose positron emission tomography
FISH: Fluorescence in situ hybridization
LGFMS: Low‐grade fibromyxoid sarcoma
SUVmax: Standardized uptake value
SEF: Sclerosing epithelioid fibrosarcoma
TBB: Transbronchial biopsy

## 1 Introduction

Low‐grade fibromyxoid sarcoma (LGFMS) and sclerosing epithelioid fibrosarcoma (SEF) are both rare fibroblastic sarcomas that typically arise in soft tissues. Histologically, LGFMS is characterized by alternating collagenous and myxoid areas, deceptively bland spindle cells with a whorled growth pattern, and collagen rosettes. In contrast, SEF is defined by epithelioid fibroblasts arranged in cords and nests and embedded in a densely hyalinized stroma. Both tumors frequently express MUC4. LGFMS most commonly harbors *FUS::CREB3L2* or *FUS::CREB3L1* fusion genes, whereas SEF typically shows *EWSR1::CREB3L1* fusion. Clinically, LGFMS has a relatively indolent course, whereas SEF follows a more aggressive course; thus, they are generally regarded as distinct entities. However, some cases exhibit histological features of both LGFMS and SEF and are recognized as hybrid LGFMS/SEF [1, 2, 3].

Hybrid LGFMS/SEF, like LGFMS and SEF, most commonly arises in soft tissues of the extremities but has also been reported in the abdomen/retroperitoneum, trunk, bone, kidney, lung, and other visceral organs [2, 3, 4, 5, 6, 7, 8, 9, 10, 11, 12, 13, 14]. Here, we report, to our knowledge, the first case of hybrid LGFMS/SEF arising from the bronchial wall and discuss its clinicopathological and molecular features.

## 2 Clinical Summary

A Japanese woman in her 40 s with no history of malignancy underwent chest radiography during a routine health screening, which revealed an abnormal shadow. She had a history of smoking. Her family history was unremarkable.

**Figure 1:**
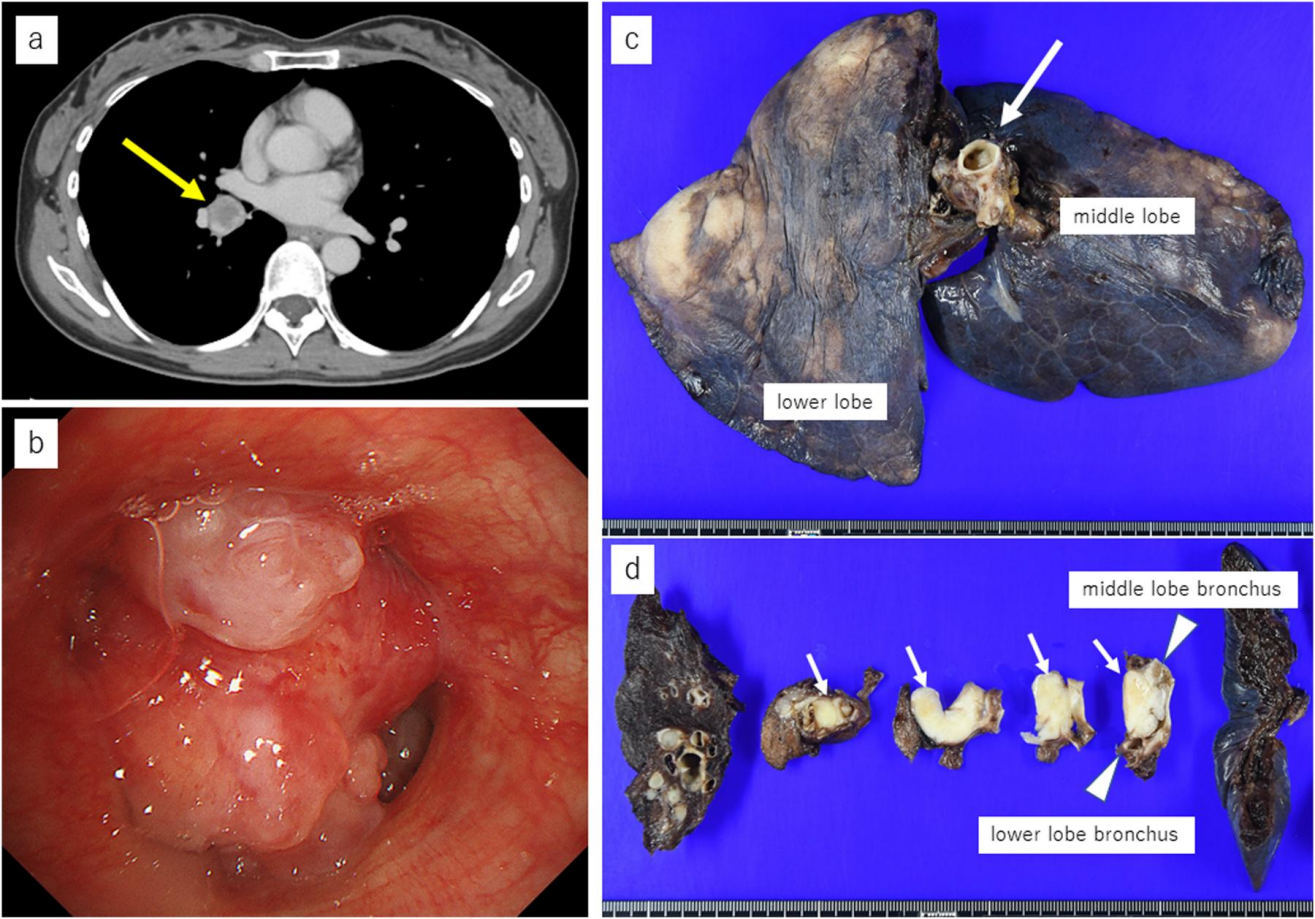
Clinical imaging and gross findings. (a) Contrast‐enhanced CT showed a low‐density mass with peripheral enhancement in the right hilar region (arrow). (b) Bronchoscopy revealed a tumor protruding into the bronchial lumen. (c) Gross appearance of the right middle and lower lobectomy specimen. A tumor protruding into the bronchial lumen was identified at the cut end of the right bronchus (arrow). (d) The cut surface revealed a 2‐cm gray‐white mass (arrow) located at the bifurcation of the middle and lower lobe bronchi (arrowhead). Mucus plugs were present in the peripheral lower bronchi.

Computed tomography (CT) revealed a mass in the right hilar region. Retrospective review of prior imaging demonstrated that the lesion had been present for at least 3 years. Contrast‐enhanced CT showed a 2‐cm low‐density mass with peripheral enhancement (Figure 1a). Fluorodeoxyglucose positron emission tomography (FDG‐PET) demonstrated mild uptake (SUVmax, 3.0) without abnormal uptake elsewhere. Based on the imaging findings, adenoid cystic carcinoma was considered the most likely diagnosis, with carcinoid tumor and squamous cell carcinoma included in the differential diagnosis. Bronchoscopy revealed a tumor protruding into the bronchial lumen, and primary bronchogenic cancer was clinically suspected (Figure 1b).

Histological examination of the transbronchial biopsy (TBB) specimen was interpreted as showing inflammatory change. However, the mass exhibited slow but progressive growth. Because malignancy could not be excluded and lower lobe atelectasis had worsened, a right middle and lower lobectomy was performed. The bronchial margin was negative on intraoperative frozen section. Postoperative thallium scintigraphy demonstrated no abnormal uptake elsewhere, supporting a primary bronchial origin. The patient remains free of recurrence or metastasis on imaging 6 months after surgery and has received no adjuvant therapy.

### 2.1 Pathological Findings

**Figure 2:**
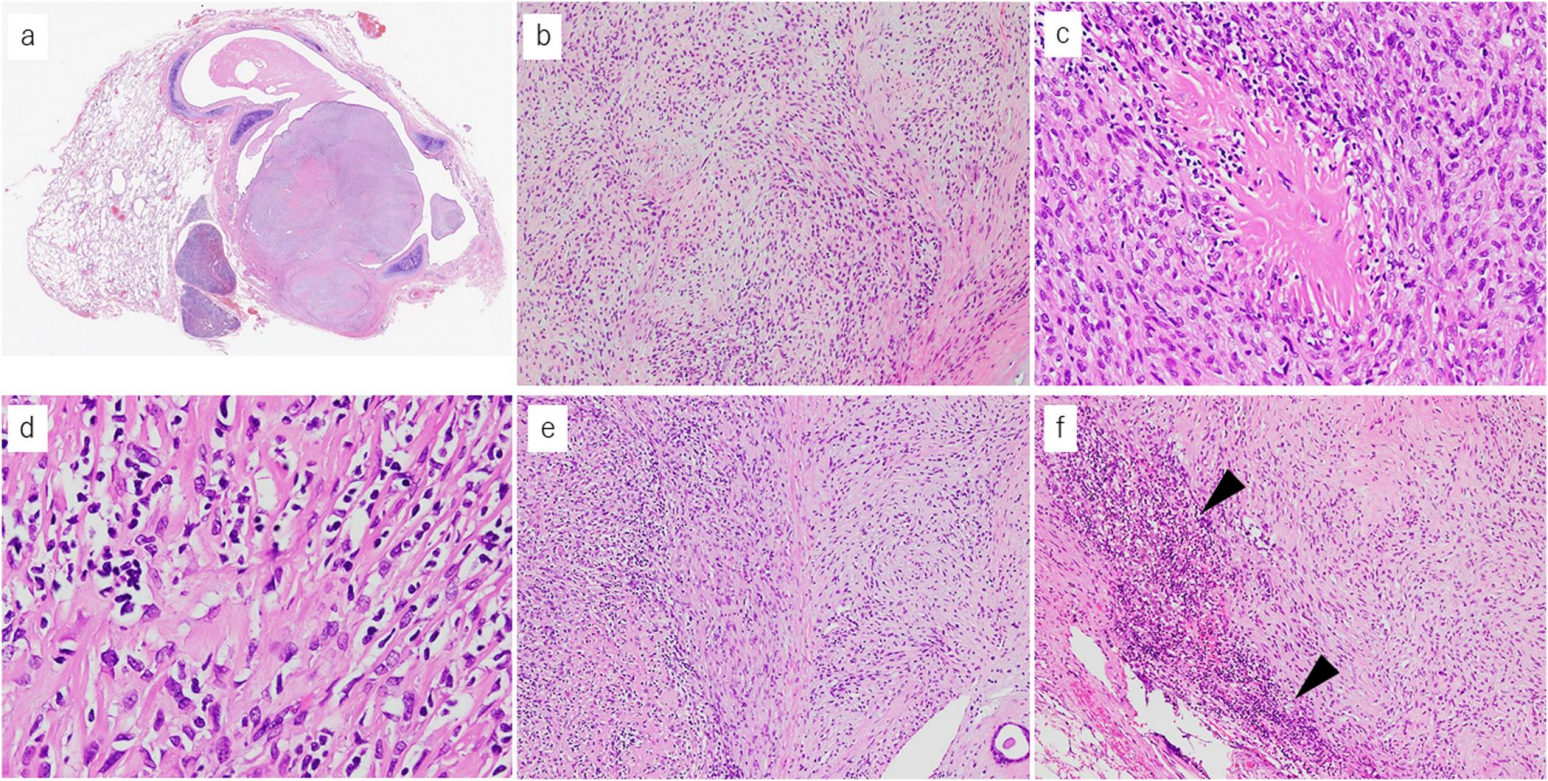
Histological findings. (a) Low‐magnification view showing a polypoid tumor projecting into the bronchial lumen from the bronchial wall. (b) The tumor was composed predominantly of poorly cohesive spindle cells arranged in fascicular and whorled patterns within a fibrous and myxoid stroma. (c) Collagen rosettes surrounded by tumor cells were observed. (d) Epithelioid cells with clear cytoplasm arranged in cords. (e) Transition between SEF and LGFMS. components (f) Direct extension of the LFGMS component into and adjacent hilar lymph node (arrowhead).

Grossly, a 2‐cm gray‐white, firm tumor was located around the bifurcation of the middle and lower lobe bronchi. Mucus plugs were present in the peripheral lower bronchi (Figure 1c,d). Histologically, the tumor was centered in the bronchial wall with polypoid protrusion into the lumen and no extension into the lung parenchyma (Figure 2a). It was relatively well demarcated and composed predominantly of bland spindle cells arranged in fascicular and whorled patterns within fibrous and myxoid stroma (Figure 2b). Hypocellular areas and numerous small vessels, hemangiopericytoma‐like vessels were present. Collagen rosettes were identified (Figure 2c). Focally, epithelioid cells with clear cytoplasm formed cords and nests within densely hyalinized stroma, consistent with an SEF component (Figure 2d). Transition between the LGFMS and SEF components was observed (Figure 2e). Mitotic figures were scarce. The bronchial surface epithelium was preserved without ulceration. Lymphocytic infiltration was present mainly in the mucosa. Within the tumor, entrapped and partially destroyed bronchial cartilage and mucous glands were scattered. Focal tumor necrosis was present. The tumor showed direct extension into an adjacent hilar lymph node (Figure 2f), but no lymph node metastasis was identified.

Based on the hematoxylin and eosin findings, the differential diagnoses included myoepithelioma/myoepithelial carcinoma, inflammatory myofibroblastic tumor, solitary fibrous tumor, malignant peripheral nerve sheath tumor, synovial sarcoma, and hybrid LGFMS/SEF.

**Figure 3:**
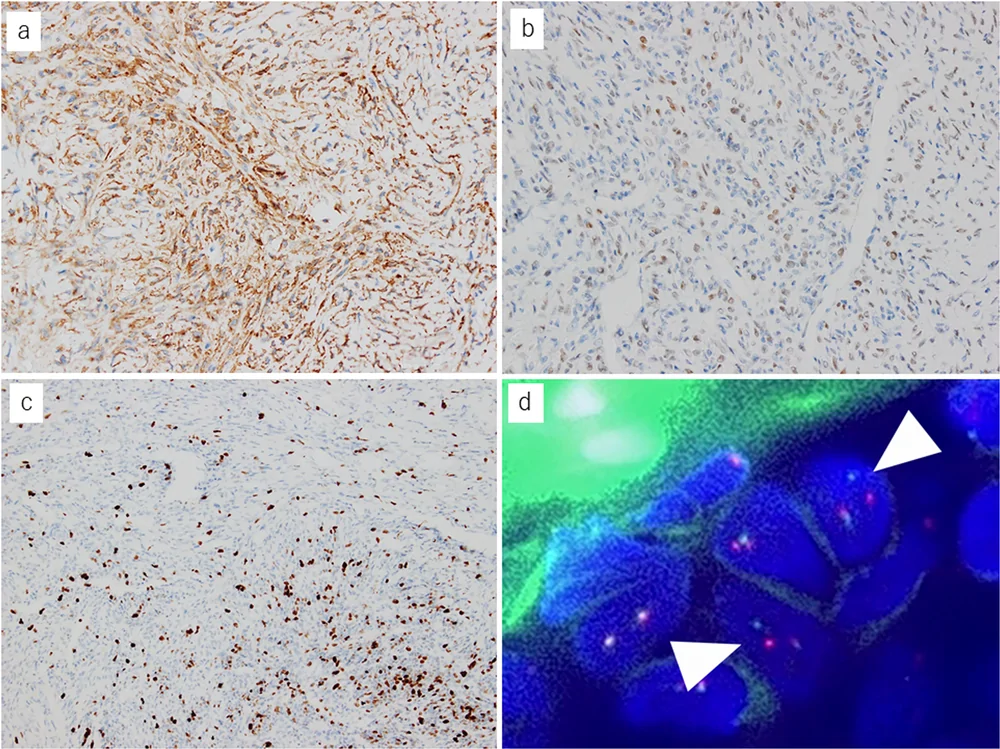
Immunohistochemical and molecular findings. (a) Immunostaining for MUC4. The tumor cells showed diffuse positivity for MUC4. (b) Immunostaining for p63. A small subset of tumor cells showed nuclear positivity for p63. (c) Immunostaining for Ki‐67. The Ki‐67 labeling index showed heterogeneous distribution. (d) FISH analysis for *EWSR1*. Tumor cells showing *EWSR1* break‐apart signals were identified (arrowhead).

Immunohistochemically, the tumor cells were diffusely positive for MUC4 (Figure 3a). p63 was positive in a small subset (Figure 3b). EMA was positive in only a few tumor cells. The tumor cells were negative for CK AE1/AE3, p40, CD34, STAT6, αSMA, S‐100, SOX10, GFAP, ALK, chromogranin, synaptophysin, ER, and PgR. SMARCB1 expression was retained, p53 showed a wild‐type pattern, and no nuclear accumulation of β‐catenin was observed. The Ki‐67 labeling index was heterogeneous, ranging from 1% to 25% (Figure 3c).

Comprehensive genomic profiling using GenMineTOP for diagnostic purposes identified an in‐frame *EWSR1::CREB3L1* fusion involving *EWSR1* exon 11 and *CREB3L1* exon 6 (Table 1). Based on the morphologic, immunohistochemical, and molecular findings, hybrid LGFMS/SEF was diagnosed.

**Table 1:** Summary of comprehensive genomic profiling findings.

Assay	GenMineTOP comprehensive genomic profiling
Tumor content	80%
Fusion detected	***EWSR1::CREB3L1***
Fusion details	*EWSR1* exon 11/*CREB3L1* exon 6, in‐frame, 2,718 supporting reads
Reported SNV/indel	*KAT6A* p.M1764V (VUS), VAF 24.4%
Copy‐number alterations	Not detected
Microsatellite instability	Microsatellite stable (MSS)
Tumor mutational burden	0.5 mutations/Mb

*Note:* The *KAT6A* p.M1764V variant was classified as a variant of uncertain significance by the molecular tumor board (MTB).

Abbreviations: Indel, insertion/deletion; MSS, microsatellite stable; SNV, single‐nucleotide variant; VAF, variant allele frequency; VUS, variant of uncertain significance.

Retrospective examination of the TBB specimen revealed immunohistochemical MUC4‐positive spindle cells within the bronchial mucosa. Fluorescence in situ hybridization (FISH) demonstrated EWSR1 break‐apart signals (Figure 3d), confirming that the biopsy had already contained tumor cells of the subsequently diagnosed hybrid LGFMS/SEF.

## 3 Discussion

LGFMS and SEF are ultra‐rare sarcomas, each with an estimated incidence of < 1 per 1,000,000 individuals [15], and hybrid LGFMS/SEF appears to be even rarer. Eleven cases of pulmonary parenchymal LGFMS [16, 17], two cases of pulmonary SEF [18], and only one case of pulmonary hybrid LGFMS/SEF [11] have been reported. Among the 67 previously reported cases of hybrid LGFMS/SEF summarized in Table 2, six arose in visceral organs, including two cases each in the kidney and small intestine and one case each in the stomach and lung.

**Table 2:** Reported cases of hybrid low‐grade fibromyxoid sarcoma/sclerosing epithelioid fibrosarcoma (LGFMS/SEF).

Reference (no. of cases)	Age, years	Sex (M/F)	Primary site	Organ category	Collagen rosettes	Molecular analysis	Metastasis	Outcome
Doyle et al. 2012 [3] *N* = 12	NR (*n* = 12)	NR (*n* = 12)	NR (*n* = 12)	NR (*n* = 12)	NR (*n* = 12)	*FUS* rearrangement (*n* = 2), *EWSR1::CREB3L1* (*n* = 2), NR (*n* = 8)	NR (*n* = 12)	NR (*n* = 12)
Ferreira et al. 2013 [4] *N* = 1	53	F	Axillary fossa	Soft tissue	Positive	NR	Lung and pleura	AWD
Wojcik et al. 2014 [5] *N* = 2	43–66 (55)	M (*n* = 2)	C6 vertebra (*n* = 1), rib (*n* = 1)	Bone (*n* = 2)	NR (*n* = 2)	*FUS* rearrangement (*n* = 1), Negative (*n* = 1)	No (*n* = 2)	NFU (*n* = 2)
Prieto‐Granada et al. 2015 [6] *N* = 8	18–75 (47)	6/2	Thigh (*n* = 4), buttock (*n* = 1), penis (*n* = 1), paraspinal (*n* = 1), neck (*n* = 1)	Extremity (*n* = 4), trunk (*n* = 4)	Positive (*n* = 3), NR (*n* = 5)	*FUS::CREB3L2* (*n* = 8)	Lung (*n* = 5), Liver (*n* = 1), No metastasis (*n* = 2)	NED (*n* = 3), AWD (*n* = 2), DOD (*n* = 3)
Mok et al. 2018 [7] *N* = 1	10	F	Kidney	Visceral organ	NR	*EWSR1::CREB3L1*	Dissemination	DOD
Sambri et al. 2018 [8] *N* = 2	17–30 (24)	1/1	Thigh (*n* = 2)	Extremity (*n* = 2)	NR (*n* = 2)	NR (*n* = 2)	Lung and bone (*n* = 1), No metastasis (*n* = 1)	NED (*n* = 1), DOD (*n* = 1)
Murshed et al. 2020 [9] *N* = 1	48	M	Small intestine	Visceral organ	NR	*HEY1::NCOA2*	Liver and bone	NFU
Kosemehmetoglu et al. 2021 [10] *N* = 4	15–60 (40)	2/2	Rib (*n* = 1), Vertebra (*n* = 1), Femur (*n* = 1), Mandible (*n* = 1)	Bone (*n* = 4)	Positive (*n* = 3), NR (*n* = 1)	*FUS* rearrangement (*n* = 1), NR (*n* = 3)	No metastasis (*n* = 4)	AWD (*n* = 4)
Lavin et al. 2023 [11] *N* = 1	11	F	Lung	Respiratory tract	NR	*EWSR1::CREB3L1*	No metastasis	NED
Suster et al. 2024 [12] *N* = 3	16–47 (30)	1/2	Femur (*n* = 1), Clavicle (*n* = 1), Temporal bone (*n* = 1)	Bone (*n* = 3)	Positive (*n* = 2), NR (*n* = 1)	*EWSR1::CREB3L1* (*n* = 3)	No metastasis (*n* = 3)	NED (*n* = 2), NFU (*n* = 1)
Alkameshki et al. 2024 [13] *N* = 1	38	M	Thigh	Extremity	Positive	*FUS::CREB3L2*	Lung	AWD
Giani et al. 2025 [2] *N* = 23	30–58 (45)	11/12	NR (*n* = 23)	Extremity (*n* = 11), abdomen/retroperitoneum (*n* = 5), trunk (*n* = 3), other (*n* = 4)	NR (*n* = 23)	*FUS::CREB3L2* (*n* = 3), *FUS* rearrangement, no fusion partner specified (*n* = 5), *EWSR1::CREB3L1* (*n* = 4), *EWSR1* rearrangement, no fusion partner specified (*n* = 1), Negative (*n* = 4), NR (*n* = 6)	Positive (*n* = 8), Negative (*n* = 15)	NED (*n* = 17), AWD (*n* = 2), DOD (*n* = 2), NFU (*n* = 2)
Ahsan et al. 2026 [14] *N* = 8	30–66 (41)	6/2	Retroperitoneum (*n* = 2), retroperitoneum/kidney (*n* = 1), omentum (*n* = 1), stomach (*n* = 1), paravertebral/lumbar (*n* = 1), abdominal wall (*n* = 1), mesentery and small intestine (*n* = 1)	Retroperitoneum (*n* = 3), visceral organ (*n* = 3), abdomen (*n* = 2), trunk (*n* = 1)	NR (*n* = 8)	*FUS::CREB3L2* (*n* = 3), *EWSR1::CREB3L1* (*n* = 1), *EWSR1::CREB3L2* (*n* = 1), NR (*n* = 3)	Lung and soft tissue (*n* = 1), Systemic (*n* = 1), No metastasis (*n* = 6)	AWD (*n* = 3), NFU (*n* = 5)
This case	48	F	Bronchial wall	Respiratory tract	Positive	*EWSR1::CREB3L1*	No metastasis	NED

Abbreviations: AWD, alive with disease; DOD, died of disease; F, female; M, male; NED, no evidence of disease; NFU, no follow‐up; NR, not reported.

Lavin et al. reported a pulmonary hybrid LGFMS/SEF in an 11‐year‐old girl harboring an *EWSR1::CREB3L1* fusion, as in our case [11]. The patient presented to the emergency department with a 1‐day history of cough, shortness of breath, and three episodes of bright red hemoptysis. The tumor presented as a 4.9 × 3.0 cm mass in the peripheral right lung with coarse calcification, and pulmonary hamartoma was considered the most likely diagnosis based on the imaging findings. The tumor was diagnosed and treated using robotic‐assisted bronchoscopy, suggesting a possible airway location. However, detailed histopathological findings regarding its precise site of origin were not provided, and no histological images were presented. Therefore, although an airway location is possible, a bronchial wall origin cannot be established from the available information. In contrast, the present tumor was a 2‐cm centrally located mass without calcification. Based on the imaging findings, tumors that more commonly arise from the bronchial wall, such as adenoid cystic carcinoma and carcinoid tumor, were considered the leading differential diagnoses. Histologically, the tumor was centered in the bronchial wall, with polypoid protrusion into the bronchial lumen and no extension into the lung parenchyma, clearly establishing its bronchial wall origin. Thus, to our knowledge, the present case represents the first clinicopathologically characterized hybrid LGFMS/SEF arising from the bronchial wall.

Hybrid LGFMS/SEF contains varying proportions of conventional LGFMS and characteristic SEF areas [6, 12]. In the present tumor, typical LGFMS areas with bland spindle cells, fibromyxoid stroma, and collagen rosettes transitioned into areas of epithelioid cells arranged in cords with densely hyalinized stroma, supporting the morphologic diagnosis of hybrid LGFMS/SEF. Collagen rosettes have been reported in approximately 30% of LGFMS [1]. Among the 67 previously reported cases of hybrid LGFMS/SEF, collagen rosettes were explicitly documented in 10 cases, whereas their presence or absence was not described in the remaining 57 cases (Table 2). None of the previous reports stated that collagen rosettes were absent. Although these findings suggest that collagen rosettes may be a relatively common histological feature of hybrid LGFMS/SEF, the available data are insufficient to determine their true frequency because few reports have specifically addressed this feature.

In cases of hybrid LGFMS/SEF in which collagen rosettes are inconspicuous or absent, the differential diagnoses include solitary fibrous tumor, inflammatory myofibroblastic tumor, malignant peripheral nerve sheath tumor, and synovial sarcoma. Previous studies have reported p63 immunoreactivity in hybrid LGFMS/SEF [4], and a subset of tumor cells in the present case also showed immunohistochemical positivity for p63. Therefore, myoepithelial tumors should also be considered an important differential diagnosis. In this context, MUC4 immunohistochemistry is particularly valuable in supporting the diagnosis of hybrid LGFMS/SEF.

LGFMS is generally regarded as an indolent tumor, whereas SEF is more aggressive, and hybrid LGFMS/SEF has been reported to behave more like SEF [1, 2]. Among the 11 previously reported cases of primary pulmonary LGFMS, only one case, which showed dedifferentiation, developed metastases to the pancreas and mediastinum [16, 17]. Both reported cases of primary pulmonary SEF developed metastases [18]. In contrast, no recurrence or metastasis has been reported in hybrid LGFMS/SEF of the respiratory tract [11], including the present case. However, among the 67 previously reported cases of hybrid LGFMS/SEF, seven patients with metastases died of disease (Table 2). Marked pleomorphism, necrosis, and prominent mitotic activity are unusual in LGFMS and SEF. The present tumor showed no marked nuclear atypia or pleomorphism and no increased mitotic activity but exhibited focal tumor necrosis and a Ki‐67 labeling index reaching 25% in hot spots. Focal necrosis has been reported in four of eight hybrid tumors in one previous study [6], indicating that its presence is not inconsistent with hybrid LGFMS/SEF. Ki‐67 indices have been documented in only four cases, with values of 1%, 10%, and 5%–10% in two cases [4, 10]. Thus, the present tumor contained areas with a higher Ki‐67 labeling index than previously reported, which may indicate increased proliferative activity, although its prognostic significance remains uncertain. The tumor also directly extended into an adjacent hilar lymph node; however, this represented contiguous extension rather than nodal metastasis, and no lymph node or distant metastases were identified. Given the limited literature, it is difficult to predict the prognosis of the present case based on these findings. The patient remains free of recurrence or metastasis 6 months after surgery without adjuvant therapy; however, the short follow‐up period warrants careful long‐term surveillance.

LGFMS commonly harbors *FUS* gene rearrangements, whereas SEF more frequently shows *EWSR1* gene rearrangements. In the multicenter retrospective study by Giani and colleagues, *FUS* rearrangements and *EWSR1* rearrangements were identified in 66.1% and 0.8% of LGFMS cases, respectively; in 6.3% and 53.1% of SEF cases, respectively; and in 73.9% and 39.1% of hybrid LGFMS/SEF cases, respectively [2]. The most common fusions in hybrid LGFMS/SEF are *FUS::CREB3L2* and *EWSR1::CREB3L1*. Among visceral hybrid LGFMS/SEF, *FUS::CREB3L2* and *HEY1::NCOA2* fusions have been identified in the small intestine, *FUS::CREB3L2* in the stomach, and *EWSR1::CREB3L1* in the kidney and lung (Table 2). Although the limited available data suggest that *FUS::CREB3L2* fusion may be more frequent in tumors arising in the gastrointestinal tract, whereas *EWSR1::CREB3L1* fusion may be more frequent in tumors arising in the respiratory tract, the number of reported cases arising in visceral organs remains too small to establish an association between fusion status and anatomic site. In the present case, *EWSR1::CREB3L1* fusion further supported the morphologic diagnosis of hybrid LGFMS/SEF. However, the biological and clinicopathological significance of *FUS*‐rearranged versus *EWSR1*‐rearranged hybrid LGFMS/SEF remains unclear. Further accumulation of cases is therefore needed to clarify the clinicopathological significance of fusion status, including its potential associations with the site of origin and clinical course.

Furthermore, novel MUC4‐positive fibrosarcoma associated with APC alterations or *EPS15::KLF17*/*EPS15L1::KLF17* fusions have recently been described as entities distinct from the LGFMS‐SEF spectrum [19, 20], emphasizing the increasing importance of molecular analysis in classifying MUC4‐positive fibroblastic sarcomas.

The present case also illustrates the difficulty of diagnosing hybrid LGFMS/SEF in small biopsy specimens. Although the TBB had sampled the tumor, neither obvious neoplastic spindle cell proliferation nor characteristic collagen rosettes or epithelioid fibroblasts were recognized initially. Because hybrid LGFMS/SEF is histologically heterogeneous, the characteristic SEF component may be absent in limited biopsy specimens. In a previously reported case, pulmonary hamartoma was also considered in the differential diagnosis of pulmonary hybrid LGFMS/SEF on biopsy [11]. Furthermore, when a MUC4‐positive spindle cell tumor is identified, evaluation for relevant gene alterations by FISH or next‐generation sequencing is important.

In conclusion, we describe the first case of hybrid LGFMS/SEF arising from the bronchial wall. Because hybrid LGFMS/SEF arising in the respiratory tract is extremely rare, many aspects of its pathogenesis, biological behavior, prognostic factors, and optimal treatment strategies remain unclear. Further accumulation of cases and comprehensive clinicopathologic and molecular analyses are needed to better define the biological behavior and optimal management of this exceptionally rare tumor.

## Author Contributions

Hiroko Ikeda performed the histopathological evaluation and drafted the manuscript. Seiichi Kakegawa, Daisuke Saito, and Seiji Yano evaluated the clinical data. Keishi Mizuguchi, Akihiro Nishiyama, and Shinji Takeuchi performed the genetic analyses. Ayumi Ito and Keita Kinoshita contributed to the histopathological evaluation. Tomohiro Kobayashi and Satoshi Kobayashi evaluated the radiological findings. Isao Matsumoto supervised the study. All authors reviewed and approved the final manuscript.

## Funding

The authors have nothing to report.

## Ethics Statement

All procedures were performed in accordance with the Declaration of Helsinki. Written informed consent was obtained from the patient. Patient anonymity has been preserved.

## Conflicts of Interest

The authors declare no conflicts of interest.

## Data Availability

The data that support the findings of this study are available from the corresponding author upon reasonable request.
